# Lactoferrin as Protective Natural Barrier of Respiratory and Intestinal Mucosa against Coronavirus Infection and Inflammation

**DOI:** 10.3390/ijms21144903

**Published:** 2020-07-11

**Authors:** Elena Campione, Terenzio Cosio, Luigi Rosa, Caterina Lanna, Stefano Di Girolamo, Roberta Gaziano, Piera Valenti, Luca Bianchi

**Affiliations:** 1 Dermatologic Unit, Department of Systems Medicine, University of Rome Tor Vergata, 00133 Rome, Italy; terenziocosio@gmail.com (T.C.); caterinalanna.cl@gmail.com (C.L.); luca.bianchi@uniroma2.it (L.B.); 2 Department of Public Health and Infectious Diseases, University of Rome La Sapienza, 00185 Rome, Italy; luigi.rosa@uniroma1.it (L.R.); piera.valenti@uniroma1.it (P.V.); 3 Department of Otorhinolaryngology, University of Rome Tor Vergata, 00133 Rome, Italy; stefano.di.girolamo@uniroma2.it; 4 Department of Experimental Medicine, University of Rome Tor Vergata, 00133 Rome, Italy; roberta.gaziano@uniroma2.it

**Keywords:** coronavirus, SARS, lactoferrin

## Abstract

Recently, the world has been dealing with a devastating global pandemic coronavirus infection, with more than 12 million infected worldwide and over 300,000 deaths as of May 15th 2020, related to a novel coronavirus (2019-nCoV), characterized by a spherical morphology and identified through next-generation sequencing. Although the respiratory tract is the primary portal of entry of SARS-CoV-2, gastrointestinal involvement associated with nausea, vomiting and diarrhoea may also occur. No drug or vaccine has been approved due to the absence of evidence deriving from rigorous clinical trials. Increasing interest has been highlighted on the possible preventative role and adjunct treatment of lactoferrin, glycoprotein of human secretions part of a non-specific defensive system, known to play a crucial role against microbial and viral infections and exerting anti-inflammatory effects on different mucosal surfaces and able to regulate iron metabolism. In this review, analysing lactoferrin properties, we propose designing a clinical trial to evaluate and verify its effect using a dual combination treatment with local, solubilized intranasal spray formulation and oral administration. Lactoferrin could counteract the coronavirus infection and inflammation, acting either as natural barrier of both respiratory and intestinal mucosa or reverting the iron disorders related to the viral colonization.

## 1. Introduction

Recently, the world has been dealing with a devastating global pandemic coronavirus infection, with more than 45 million infected worldwide and over 300,000 deaths as of May 15th 2020, related to a novel coronavirus (2019-nCoV), characterized by a spherical morphology and identified through next-generation sequencing. The genetic sequence of SARS-CoV-2 shows more than 80% identity to SARS-CoV and 50% to the MERS-CoV [1,2]. Of note, SARS-CoV-2 possesses a higher transmissibility from human to human and lower pathogenicity in respect to SARS-CoV [3].

Coronavirus belongs to the family of *Coronaviridae*, subfamily *Coronavirinae*, order *Nidovirales* and this subfamily includes four genera: *Alphacoronavirus*, *Betacoronavirus, Gammacoronavirus*, and *Deltacoronavirus* [4]. Coronavirus possesses a single-strand, positive-sense RNA genome ranging from 26 to 32 kilobases in length [5]. The first open reading frame represents the majority of the viral genome and encodes 16 non-structural proteins, while the other open reading frames encode structural and accessory proteins [6,7]. The residual viral genome is responsible for the expression of four essential structural proteins: spike glycoprotein, small envelope protein, matrix protein, and nucleocapsid protein. In particular, spike (S) glycoprotein is composed of two subunits (S1 and S2) [6]. Homotrimers of S proteins on the viral surface are responsible for binding to host receptors (S1) and membrane fusion (S2) [2,8]. Noteworthy, S1 subunit, like in other Beta-coronaviruses, is composed of a core and an external subdomain and represents only a 40% amino acid identity with other SARS-CoVs. However, in SARS-CoV-2, the S2 subunit, which contains a fusion peptide, a transmembrane domain and a cytoplasmic domain, is highly conserved. S1 directly interacts with angiotensin-converting enzyme 2 (ACE-2), the functional receptor expressed on the surface of pulmonary, cardiac, renal, intestinal and endothelial host cells [2]. In particular, alveolar epithelial parenchymal type II cells express ACE-2. Notably, nasal epithelial cells, comprising two clusters of goblet cells and one cluster of ciliated cells, show the highest expression among all investigated cells in the respiratory tree [9]. As the virus can be detected in upper respiratory tract samples, the nasofarinx can be involved as a site of replication. Furthermore, SARS-CoV-2 is also able to infect T lymphocytes, despite their very low expression levels of ACE-2, leading researchers to hypothesize the presence of an alternative receptor allowing viral entry into these cells [10]. In addition, studies have shown that the serine protease TMPRSS2 can prime S protein, thus allowing spike protein cleavage and regulating the entire mechanism of viral entry [11]. Other proteases can also be involved [11]. The serine protease TMPRSS2 and cathepsin B/L, expressed by salivary glands, lung, small intestine, liver, kidney, and heart endothelial cells, could lead as a consequence to systemic vasculitis, thromboembolism and disseminated intravascular coagulation [12]. Further hypothetical targets could consider other viral components such as ORF3b, not homologous with that of SARS-CoVs, and a secreted protein (encoded by ORF8), structurally different from those of SARS-CoV [4]. 

Although the respiratory tract is the primary portal of entry of SARS-CoV-2, gastrointestinal involvement associated with nausea, vomiting and diarrhoea and persistence of viral particles may also occur [4,10]. Early studies found a low incidence (1–3.5%) of gastrointestinal or hepatic manifestations, but more recent studies reported a higher rate of affection (11.4–24.2%). Moreover, in subjects suffering from SARS-CoV-2, transaminases may range from mild to elevated levels [13], probably related to the presence of ACE-2 receptors on enterocytes in the ileum and colon [14], cholangiocytes, and hepatocytes [15,16,17]. ACE-2 seems to mediate inflammatory processes and, consequently, the occurrence of diarrhoea [18]. As several studies indicated a possible faecal–oral transmission, SARS-CoV-2 RNA should be detected in the stool of patients affected by Covid-19 [19]. However, it is unclear whether SARS-CoV-2 replicates in human intestine and contributes to possible faecal–oral transmission [18] or the intestine is a potential site of SARS-CoV-2 replication, thus contributing to local and systemic illness and overall disease progression [10]. Gut dysbiosis secondary to gastrointestinal inflammation is known to possibly interfere with distant disorders [20]. In light of this view, a gut–lung axis, bidirectionally acting, has been hypothesized: endotoxins and microbial metabolites synthesized by gut microbiota can influence the lung through the circulation, while lung inflammation can affect the gut microbiota [21]. Therefore, gut microbiota could participate in the pathogenesis of acute respiratory distress syndrome and vice versa [22].

Most patients infected with SARS-CoV-2 exhibit mild-to-moderate symptoms, such as sudden anosmia or ageusia, fever, abnormal cough, headache and fatigue, and diarrhoea, and recover without sequelae. However, around 15% develop severe pneumonia and 5% progress to acute respiratory distress syndrome, septic shock, and/or multiple organ failure, associated with high mortality.

## 2. Iron and Inflammatory Homeostasis

Among several factors affecting viral infections as well as the related inflammatory processes, iron plays a critical role, by favouring viral progression on one side and exacerbating inflammatory processes on the other. Indeed, iron is a pivotal element for all cells as it is a fundamental in DNA replication and energy production in both humans and microorganisms. When iron is present in excess, it generates reactive oxygen species (ROS), causing its ability to release electrons to oxygen. ROS formation damages proteins, lipid membranes and DNA, causing tissue injury and organ failure [23]. In healthy conditions, free available iron is present at the concentration of about 10^−18^ M, very far from that requested for microbial multiplication, ROS generation and inflammatory process induction [24,25]. In pathological conditions, the concentration of free iron is higher than 10^−18^ M, thus increasing host susceptibility to infections, ROS and inflammatory process induction [25]. The correct balance of iron between tissues/secretions and blood, defined as iron homeostasis, involves several iron proteins, such as transferrin (Tf), ferroportin (Fpn), ferritin (Ftn), lactoferrin (Lf) as well as hepcidin, an important peptide synthetized by liver [25]. During viral infections, iron homeostasis is perturbed, leading to iron disorders [26] which are worsened by the action of pro-inflammatory cytokines, including interleukin-6 (IL-6) [27]. Indeed, infections and related inflammatory processes, by up-regulating IL-6, induce hepcidin synthesis, which, in turn, blocks Fpn-mediated iron efflux from the cells to blood, thus reducing serum iron concentration and increasing iron overload in reticuloendothelial cells [28]. Consistently, when serum IL-6 level increases, the iron-saturation of serum Tf, as well as levels of Tf receptor 1 (TfR1) and Fpn, decrease [27,28]. Therefore, the host’s iron status can alter the course of infection and its resolution. Moreover, viral infections need active cell metabolism and, therefore, a significant viral replication requires a high iron availability [28,29]. As a matter of fact, iron homeostasis disorders including Fpn, TfR1 and Ftn dysregulated synthesis lead to an intracellular iron overload which facilitate viral multiplication and spreading.

Therefore, iron dysregulation, induced by infection by SARS-CoV-2 and related inflammatory processes, could also play a crucial role in the activation and progression of organs impairment. 

In patients affected by COVID-19, most of the severe cases demonstrated massive systemic levels of infection-related biomarkers and inflammatory cytokines, namely serum IL-6, tumour necrosis factor-α (TNFα) and ftn [30]. The excessive release of pro-inflammatory cytokines, referred as a ‘cytokine storm’, has evolved as an important surveillance system that, when triggered, fights infection and eliminates pathogens but may contribute as a principle contributory cause of internal organs or systems impairment as recently hypothesized for cardiovascular, neurological, septic shock or cutaneous sequelae or related risk factors for poor prognosis [31,32,33,34,35,36,37,38,39,40].

## 3. Therapeutic Options for Covid-19 

No drug or vaccine against SARS-CoV-2 has been approved due to the absence of evidence deriving from rigorous clinical trials. Moreover, although some treatments seem to be effective against this virus, they exert several adverse effects. Safe and effective drugs are needed to prevent and cure coronavirus disease 2019 (COVID-19). 

In light of these considerations, alongside the need to find specific safe antiviral agents or vaccines, it would be of crucial importance increase the host’s defences by creating an immunological barrier able to protect the upper respiratory tract, considered the main access route of the virus into the host, or/and the gut mucosa. It is a noteworthy observation that a consistent number of COVID-19 patients present diarrhoea and more often olfactory and gustatory signs, even with no or mild respiratory symptoms and a normal otolaryngologic exam. In symptomatic and asymptomatic patients, nasal swabs have yielded higher viral loads than throat swabs, implicating the nasal epithelium as a portal for initial infection and transmission [41], whereas several researchers are testing for evidence of the virus in the stool.

Of note, among different therapies, convalescent plasma containing specific antibodies against SARS-CoV-2 can be applied as a main treatment [15] according to the National Health Commission of the People’s Republic of China guideline (National Health Commission of the People’s Republic of China. 2020. Notice on printing and distributing the convalescent plasma treatment for novel coronavirus pneumonia (trial version 2)) [42]. However, the evaluation of the efficacy of convalescent plasma treatment is still uncertain and clinical trials are in progress. Promising results have been found through a therapy with monoclonal antibodies, the major class of biotherapeutics for passive immunotherapy, already exerting an interesting efficacy in neutralizing SARS-CoV and MERS-CoV infection [10]. However, the large-scale production of monoclonal antibodies is labour-intensive, expensive, and time-consuming. Vaccination, based on inactivated vaccines, recombinant subunits vaccines, nucleic acid-based vaccines, adenoviral vector vaccines, and recombinant influenza viral vector vaccines, is the most efficient strategy to prevent and control COVID-19, especially utilizing as a target the S protein [43]. Moreover, a screened set of SARS-CoV-derived B cell and T cell epitopes have been characterized and would be pivotal for the initial phase of vaccine development [44]. In addition, to provide reliable results on the efficacy of convalescent plasma and vaccines, several drugs to decrease the hyper inflammatory processes during COVID-19 and/or inhibit SARS-CoV-2 entry and fusion have been proposed. As a matter of fact, in some patients infected with SARS-CoV-2, a dysregulation of the immune response leads to a hyper inflammatory condition. Higher percentages of granulocyte-macrophage colony-stimulating factor-positive (GM-CSF^+^) and IL-6^+^ CD4^+^ T cells have been observed on patients in intensive care units (ICUs) compared with non-ICU patients with COVID-19, thus indicating that the cytokine storm is associated with disease severity [30]. The inhibition of excessive inflammatory response by corticosteroids may represent an additional therapy for COVID-19, even if their efficacy against this disease is still controversial [45]. Putative antiviral molecules are focused on structural CoVs components, mostly represented by the two spike subunits on the viral surface, regulating the binding to host receptors. Other drugs inhibit SARS-CoV-2 entry. As already reported, S1 protein of SARS-CoV-2 mediates the entry into host cells [46] through the binding with ACE-2 receptor [41]. However, it has been suggested that SARS-CoV-2 entry depends not only on ACE-2 but also on the host cell serine protease TMPRSS2 [11]. SARS-CoV-2 binds to ACE-2 receptor through the N-terminal S1 subunit [11,47,48], which is subsequently cleaved by the host transmembrane serine protease 2 (TMPRSS2) to expose the C-terminal S2 subunit that induces virus-cell fusion [11,49]. An inhibitor of TMPRSS2, camostat mesylate, seems to significantly reduce in vitro lung cell line infection with SARS-CoV-2, thus suggesting a putative treatment against COVID-19 [11]. In addition, it has been also suggested that the treatment with arbidol is able to inhibit virus entry. Concerning the treatment with chloroquine, a traditional antimalarial drug, its efficacy against SARS-CoV-2 has been demonstrated in vitro [50]. Clinical trials are in progress, even if the mechanism of action of chloroquine is still unknown, while its toxicity is well known. Finally, several antiviral agents have been developed against viral proteases, polymerases, MTases, and entry proteins [3]. Overall, immunotherapy with immune IgG antibodies combined with antiviral drugs may be an alternative treatment against COVID-19 until stronger options such as vaccines are available.

Therefore, there is an urgent need to explore alternative methods for treating, without adverse effects, COVID-19 clinically advanced conditions in order to reduce viral infection, replication and spread as well as mortality, and to mitigate the potential future outbreaks, before the set-up of vaccine. The hypothesis to identify natural molecules, which, without side effects, are able to increase the host’s local defences or to inhibit viral infection as well as to restore the iron and inflammatory homeostasis disorders, is extremely tempting. 

## 4. Lactoferrin

Recent studies have demonstrated that components of the human secretions, belonging to innate immunity, are key elements of host defences acting as a pivotal barrier against viral injury [25,51,52]. Increasing interest has been very recently highlighted on the possible preventive role and adjunct treatment of lactoferrin [53], a glycoprotein of human secretions that is part of a non-specific defensive system, known to play an important role against microbial and viral infections and exerting anti-inflammatory effects on different mucosal surfaces and able to regulate iron metabolism [25,51].

Lactoferrin (Lf), belonging to the Tf family, is able to reversibly chelate two Fe (III) per molecule with high affinity (K_d_ ~ 10^−20^ M). It is a cationic glycoprotein of ca. 690 amino acid residues. Differently from Tf, which releases iron at pH values lower that 5.5, Lf binds ferric iron until pH values of about 3.0. Human Lf (hLf), a molecule of the innate immunity, is constitutively secreted by exocrine glands and by neutrophils in infection and inflammation sites (10^6^ neutrophils release 15 µg of Lf) [25,54,55]. Even if Lf is highly conserved among different species, the highest sequence homology has been recognized between human and bovine lactoferrin (bLf) (about 70%) [56]. BLf and hLf possess identical biological functions [25] and, therefore, bLf has been applied in in vitro and in vivo studies, being generally recognized as safe (GRAS) by the Food and Drug Administration (FDA) and available in large quantities. Lf’s various functions are associated with its capacity to chelate two ferric ions and to bind to anionic surfaces [57]. Lf possesses a potent anti-inflammatory and immunomodulatory activities [48,49,58]. Lf’s anti-inflammatory activity depends on its ability to enter, through receptor-mediated endocytosis, inside host cells and to translocate into the nucleus [59], thus regulating pro-inflammatory gene expression [60,61]. Lf, through its anti-inflammatory activity and immunomodulatory properties, is also able to down-regulate pro-inflammatory cytokines and to potentiate the adaptive immune response. Moreover, Lf’s ability in counteracting and reverting iron disorders, by modulating immune response and down-regulating pro-inflammatory cytokines, such as IL-6, has been demonstrated both in in vitro [62,63] and in vivo [64,65] models, as well as in clinical trials [66,67]. Of note, Lf has been proven to act as a scavenger against iron overload and inflammation in lung epithelium of mice infected by *Pseudomonas aeruginosa* [64,65], and bLf was found to rebalance lung iron-handling proteins and to decrease broncho-alveolar iron overload, one of the main actor in infection progression and exacerbation [65]. Several studies described Lf’s antiviral activity towards enveloped and naked viruses, related to different virus families, such as *Retroviridae* (human immunodeficiency viruses), *Papillomaviridae* (human *papillomavirus*), *Herpersviridae* (*Cytomegalovirus, Herpes simplex virus*), *Caliciviridae* (*feline calicivirus*), *Flaviviridae* (hepatitis C virus, Japanese encephalitis virus) *Reoviridae* (*rotavirus)*, *Adenoviridae* (*adenovirus*), *Pneumoviridae* (respiratory syncytial virus), *Paramixoviridae* (parainfluenza virus), *Orthomixoviridae* (influenza A virus), *Hepadnaviridae* (hepatitis B virus), *Picornaviridae* (*poliovirus, enterovirus 71, echovirus 6*), *Togaviridae (Alphavirus) and Geminiviridae* (tomato yellow leaf curl virus) [68,69]. bLf has been found to hinder viral entry into host cells through its competitive binding to the cell surface receptors, mainly negatively charged compounds such as glycosaminoglycans (GAGs) [68,70,71,72,73,74,75]. In addition, Lf was found to prevent viral infections by binding to dendritic cell-specific intercellular adhesion molecule 3-grabbing non-integrin (DC-SIGN) and LDL receptors [76,77]. Overall, the antiviral effect of Lf occurs in the early phase of infection, preventing the entry of viral particles into the host cells, either by blocking cellular receptors and/or by directly binding to the viral particles. Moreover, Lf is also able to exert an antiviral activity when it is added in the post-infection phase, as demonstrated in Rotavirus infection by Superti et al. [78] and in HIV infection by Puddu et al. [79]. Lf efficacy in the post-infection phase induces us to hypothesize that this glycoprotein is also efficient in interfering with the intracellular step of virus infection.

Overall, Lf exerts its antiviral activity against the majority of the tested viruses by binding to heparan sulphate, while against few viruses by interacting with surface components of viral particles [68,69]. In particular, Lf binds to E1 and E2 proteins of HCV [80], to F protein of RSV [81] and to gp120 protein of HIV [79]. Moreover, Lf interacts against both viral particles and host cells when exerting its antiviral activity against Echovirus 6 [82]. The capability of Lf to exert antiviral activity, by binding to host cells or viral particles or both, strengthens the idea that this glycoprotein is “an important brick in the mucosal wall, effective against viral attacks” [57]. It is important to recall that Lang and colleagues investigated the role of Lf in the entry of SARS pseudovirus into Myc cells. Their results reveal that Lf was able to block the binding of the spike protein to host cells, indicating that Lf exerted its inhibitory function at the viral attachment stage [83]. However, Lf did not block the virus entry by the direct interaction of spike protein with ACE-2, the functional receptor of both SARS-CoV [83] and SARS-CoV-2 [41]. The current accepted model suggests that Lf could block viral entry by interacting with heparan sulfate proteoglycans (HSPGs), which mediate the transport of extracellular virus particles from the low affinity anchoring sites to the high affinity specific entry as ACE-2 [84]. Taken together, these results suggest that Lf could play a protective role in host defence against SARS-CoV-2 infection through binding to HSPGs, thus blocking the early interaction between SARS-CoV-2 and host cells. Furthermore, the Lf ability to enter inside the nucleus may also counteract the activation of the cytokine storm, thus avoiding systemic, lung or intestinal iron homeostasis disorders as well as disease exacerbation. Recently, the effect of bLf, in a cystic fibrosis (CF) murine model of *Pseudomonas aeruginosa* chronic lung infection has been demonstrated [65]. To induce acute or chronic lung infection, CF mice were intra-tracheal infected with multidrug-resistant MDR-RP73 *P. aeruginosa*, free or embedded in agar beads. Aerosolized bLf or saline solution treatments were performed five minutes after infection and carried out daily for six days. In infected CF mice, aerosolized bLf was found effective in significantly decreasing both pulmonary *P. aeruginosa* number as well as infiltrated leukocytes. For the first time, our results demonstrate that bLf also decreased pulmonary iron excess, in WT and CF mice. In particular, Fpn and Ftn expression was significantly decreased. Overall, the multi-functionality of bLf allows to block inflammation and iron dysbalance induced by *P. aeruginosa*, thus decreasing the severity of CF-related infection [65].

A recently investigated effect of Lf is to regulate the activation of the plasminogen, which still adds a value of this molecule in the control of coagulation cascade promoted by the virus. LF can exert negative regulatory effects on cell migration via inhibition of Plg activation and through the regulation of fibrinolysis [85]. This activity was also confirmed by evidence of a peptide with the amino acids sequence derived from lactoferrin shown antithrombotic activity [86]. 

**Figure 1:**
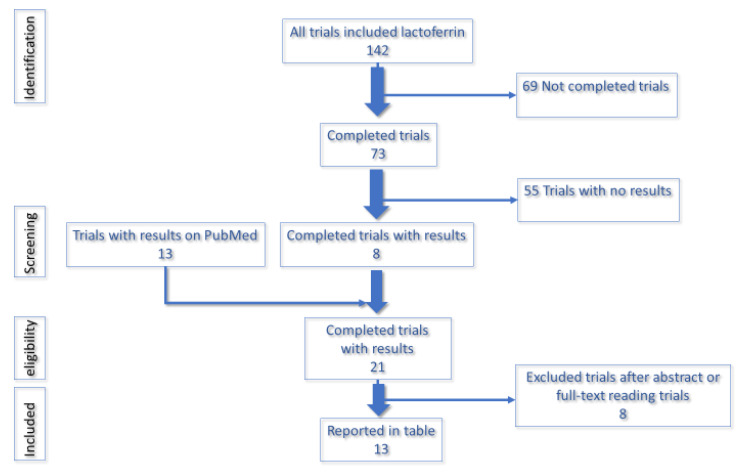
Flowchart summarizing the research strategy to select clinical trials of lactoferrin.

More than 140 trials are available on trials.gov. Among these, major contribution of lactoferrin has been demonstrated on anaemia, bacterial and viral infection, both communitarian and nosocomial, inflammation and prevention of sepsis. (Table 1). These trials assessed the safety, tolerability, efficacy of Lf both as an oral dietary supplement or as an intranasal spray. We performed a comprehensive search on US National Institutes of Health Ongoing Trials Register, using the word “lactoferrin”. The first result included 142 trials. Then, we included only “completed trials”, which gave 73. Among these 73, trials, we selected eight trials with results. In order to include results not available on US National Institutes of Health Ongoing Trials Register, we performed a cross-research on PubMed using the NTC number. This has permitted us to include another 13 completed trials with results. Among these 21 trials, eight were excluded as not pertinent to our review, analysing other drugs. Overall, 13 completed trials with results are included in the table. All human studies were included with no restrictions on age, sex, ethnicity or type of study. Trials with no results were excluded. Analyses also excluded animal studies. (Figure 1).

Taken together, all these properties pave the way to consider Lf as a promising tool able to target multi-faced aspects of the viral progression and pathogenesis in COVID-19. Historically, treatment of many lung diseases involved systemic delivery of the drug; however, this modality was generally replaced by inhalation, which is more effective, due to targeting the drug to the site of disease, and safer, reducing side effects [87,88]. In synergy, the success of novel drug delivery systems depends on the development of formulations that are capable of improving the therapeutic index of biologically active molecules by increasing their concentration specifically at desired target sites or organs. For the best therapeutic effect, it is necessary to find the best pharmacological formulation, both to block the virus in the extracellular microenvironment of the mucous membranes, and to stimulate local immunity in order to promote a better viral load clearance, considering the need to act at the level of the upper and lower airways. There is the need for a formulation with different chemical-physical characteristics. Among particular drug delivery methods used to treat lung diseases, membrane-like structures composed by triglycerides and phospholipids have been proposed. The rationale regarding the use of this drugs delivery method is based on lower toxicity and more biological compatibility to pulmonary epithelium. Alternatively, another formulation containing liposomes could be prepared with endogenous lipids, which are easier adsorbed by respiratory mucosae if intra-nasally administered [89]. Their composition can be adjusted to modulate drug release and they might encapsulate both hydrophilic and lipophilic compounds with high drug loading [90]. It is well known that the mucociliary epithelium did not offer a good surface for drug delivery. At this level, liposomes could reduce the drug clearance, improving its absorption [91,92,93]. Viral COVID-19 RNA was detected in the upper airways from symptomatic patients, with higher viral loads observed in nasal swabs compared to those obtained from the throat. Similar viral loads were observed in asymptomatic patients [41], indicating that the nasal epithelium is an important portal for initial infection, and may serve as a key reservoir for viral spread across the respiratory mucosa and an important locus mediating viral transmission. In-depth analysis of epithelial cells in the respiratory tree has revealed that nasal epithelial cells, specifically goblet/secretory cells and ciliated cells, display the highest ACE2 expression of all the epithelial cells analysed [94]. The skewed expression of viral receptors/entry-associated proteins towards the upper airway may be correlated with enhanced transmissivity. Finally, many of the top genes associated with ACE-2 airway epithelial expression are innate immune-associated, antiviral genes, highly enriched in the nasal epithelial cells. This association with immune pathways might have clinical implications for the course of infection and viral pathology, and highlights the specific significance of nasal epithelia in viral infection [94].

Taken together, we believe that all these properties justify designing a clinical trial to evaluate and verify if a local treatment of nasal mucosa with Lf solubilized in an intra-nasal spray formulation, and oral assumption of Lf, could counteract the coronavirus infection and inflammation. Lf acts either as a natural barrier of both respiratory and intestinal mucosa or reverting the iron disorders related to the viral colonization or modulating the immune response or down-regulating the pro-inflammatory cytokines released by the viral inflammation, without any risk of possible adverse events. Furthermore, the inclusion of Lf in preserving structures, such as liposomes, reduces gastric and intestinal denaturation while maintaining its integrity and therefore its biological functionality [95]. Lf could be used in asymptomatic or mildly symptomatic patients to prevent the worsening of SARS-CoV2. The Lf ideal dosage should be diversified on the basis of symptom severity. Asymptomatic COVID-19 patients should use 300 mg, orally administered, doubling the dosage (maximum 1gr) for mildly symptomatic patients [67]. We suggest maintaining the treatment at least until the COVID-19 buffer becomes negative.

## Figures and Tables

**Table 1:** Trials reported to strategy research. All trials have been checked on https://pubmed.ncbi.nlm.nih.gov/ and https://clinicaltrials.gov/ (accessed on 2 July 2020).

Official Title on ClinicalTrials.gov or Publication Title	NCT Number	Phase	Sample Size	Study Results
A Randomized Controlled Clinical Trial of Two Different Oral Care Regimens Combined With Ventilator-Associated Pneumonia (VAP) Bundle Strategy for Reduction of Duration of Mechanical Ventilation in a Neonatal Population	NCT01314742 Completed 4 February 2014	Early I	41 Randomized Parallel Assignment	https://pubmed.ncbi.nlm.nih.gov/23608625/
Safety and Efficacy of Human Lactoferrin hLF1-11 for the Treatment of Infectious Complications Among Haematopoietic Stem Cell Transplant Recipients Part A: Clinical Study Protocol SC12: Safety of a Single Dose of 5 mg of hLF1-11 Given to Autologous Haematopoietic Stem Cell Transplant Recipients	NCT00509938 Completed November, 2006	I-II	8 Non-randomized single group Assignment	https://pubmed.ncbi.nlm.nih.gov/19735580/
A Phase 2 Randomized Controlled Trial to Determine the Efficacy of Lactoferrin for the Prevention of Nosocomial Infections	NCT01996579 Completed 12 September 2016	II	214 Randomized Parallel Assignment	https://pubmed.ncbi.nlm.nih.gov/27681799/
Recombinant Lactoferrin to Reduce Immune Activation and Coagulation Among HIV Positive Patients	NCT01830595 Completed January, 2018	II	55 Randomized crossover assignment	https://pubmed.ncbi.nlm.nih.gov/30721997/
Oropharyngeal Administration of Colostrum to Extremely Low Gestational Age Newborns	NCT01536093 Completed December, 2013	II	48 Randomized Parallel Assignment	https://pubmed.ncbi.nlm.nih.gov/25624376/
Nasal Irrigation for Chronic Rhinosinusitis and Fatigue in Patients With Gulf War Illness	NCT01700725 Completed May, 2017	II	40 participants Randomized Single Group Assignment	https://pubmed.ncbi.nlm.nih.gov/25625809/
Effects of Lactoferrin on Chronic Inflammation in the Elderly	NCT02968992 Completed 25 February 2019	II	36 Randomized Parallel Assignment	https://clinicaltrials.gov/ct2/show/results/NCT02968992?term=lactoferrin&rslt=With&draw=2&rank=2
Pilot Study: Lactoferrin for Prevention of Neonatal Sepsis	NCT01264536 Completed December, 2011	II	190 Randomized Parallel Assignment	https://pubmed.ncbi.nlm.nih.gov/25973934/
Influence of Vaginal Lactoferrin Administration Prior to Genetic Amniocentesis on PGE2, MMP-9, MMP-2, TIMP-1 and TIMP-2 Amniotic Fluid Concentrations	NCT02695563 Completed September, 2015	II	190 Randomized Parallel Assignment	https://pubmed.ncbi.nlm.nih.gov/27872513/
Randomized, Controlled Trial-Lactoferrin Prevention of Diarrhea in Children	NCT00560222 Completed October, 2011	III	555 Randomized Parallel Assignment	https://pubmed.ncbi.nlm.nih.gov/22939927/
Lactoferrin for Prevention of Sepsis in Infants	NCT01525316 Completed October, 2016	III	414 Randomized Parallel Assignment	https://pubmed.ncbi.nlm.nih.gov/29613975/ https://pubmed.ncbi.nlm.nih.gov/32037149/ https://pubmed.ncbi.nlm.nih.gov/32401307/ https://pubmed.ncbi.nlm.nih.gov/28125095/ https://pubmed.ncbi.nlm.nih.gov/30743197/
Phase IV Study of Oral Administration of Bovine Lactoferrin (bLf) to Prevent and Cure Iron Deficiency (ID) and Iron Deficiency Anemia (IDA) Until Delivery in Hereditary Thrombophilia (HT) Affected Pregnant Women	NCT01221844 Completed May, 2011	IV	330 Non-randomized Parallel Assignment	https://pubmed.ncbi.nlm.nih.gov/30298070/ https://pubmed.ncbi.nlm.nih.gov/24590680/
Lactoferrin and Lysozyme Supplementation for Environmental Enteric Dysfunction	NCT02925026 Completed December, 2017	Not Applicable	235 Randomized Parallel Assignment	https://pubmed.ncbi.nlm.nih.gov/29110675/
